# Hydrothermal Ammonia Carbonization of Rice Straw for Hydrochar to Separate Cd(II) and Zn(II) Ions from Aqueous Solution

**DOI:** 10.3390/polym15234548

**Published:** 2023-11-27

**Authors:** Jiarui Wang, Xiaocheng Wei, Hao Kong, Xiangqun Zheng, Haixin Guo

**Affiliations:** 1 Agro-Environmental Protection Institute, Ministry of Agriculture and Rural Affairs, No. 31 Fukang Road, Nankai District, Tianjin 300191, China; wang_jiar@163.com (J.W.); 13752221539@163.com (X.W.); hnkonghao@163.com (H.K.); 2 Key Laboratory for Rural Toilet and Sewage Treatment Technology, Ministry of Agriculture and Rural Affairs, Tianjin 300191, China; 3 Institute of Environment and Sustainable Development in Agriculture, CAAS, Beijing 100081, China

**Keywords:** raw biomass, polymerization, hydrothermal carbonization, hydrochar yield, wastewater

## Abstract

Hydrochar is considered to be a good adsorbent for the separation of metal ions from aqueous solutions. However, the yield of hydrochar from raw straw is generally low, because the hydrothermal carbonization occurs via dehydration, polymerization, and carbonization. In this work, various hydrochar samples were prepared from rice straw with nitrogen and phosphorus salt; moreover, toilet sewage was used instead of nitrogen, and phosphorus salt and water were used to promote the polymerization and carbonization process. The modified carbon was characterized using XRD, XPS, SEM, and FTIR, and the adsorption capacity was investigated. A significant increase in hydrochar yield was observed when toilet sewage was used as the solvent in the hydrothermal carbonization process. The adsorption capacity of N/P-doped rice straw hydrochar for Cd^2+^ and Zn^2+^ metal ions was 1.1–1.4 times higher than that those using the rice straw hydrochar. The Langmuir models and pseudo-second-order models described the metal adsorption processes in both the single and binary-metal systems well. The characterization results showed the contribution of the surface complexation, the electrostatic interaction, the hydrogen bond, and the ion exchange to the extraction of Cd^2+^ and Zn^2+^ using N/P-doped rice straw hydrochar.

## 1. Introduction

Cadmium (Cd) is toxic metal and can lead to a variety of health issues among humans, such as bone damage, renal failure, neurological issues, and even cancer after prolonged exposure [1,2]. Excess zinc (Zn) can also lead to adverse health effects, such as stomach cramps, skin irritation, and nausea [3]. Cd and Zn share similar chemical properties and often appear together as water pollutants [4]. Significant concentrations of cadmium and zinc metals have been found in urban stormwater [5]. However, cadmium and Zn concentrations in drinking water should be below 0.003 mg L^−1^ and 5.0 mg L^−1^, respectively [6]. Therefore, finding effective and affordable technologies to remove Cd and Zn from wastewater has become an urgent requirement for ecological management.

Wastewater treatment technologies include adsorption technology, ion exchange, chemical precipitation, oxidation, and so on [7]. Among these methods, adsorption methods have advantages such as low cost, simple operation, and so on, which has led to their use in water treatment processes [8]. Carbon materials that have some advantages, such as low price, high surface area, unique pore structure, and so on, have been used as adsorbents in heavy metal treatment process [8,9].

Hydrothermal carbonization (HTC) is a thermochemical process that uses water as a medium; it can be used for residual management. Hydrothermal carbonization offers an efficient approach to the synthesis of biocarbon. Compared to pyrolysis carbonization processes, hydrothermal carbonization takes place at low temperatures with lower pollution emissions [8]. Hydrochar as a novel material has been used as a catalyst, adsorbent, and energy source, among other applications [10]. The substrates for HTC include fructose, glucose, cellulose, raw biomass, and so on. However, it should be noted that, compared to hydrochar from sugars, low hydrochar yields are obtained from raw biomass under the same hydrothermal carbonization conditions [11,12]. Generally, the hydrothermal process occurs via dehydration, polymerization, and carbonization reactions. Hydrochars have functional groups, a carbon skeleton, crosslinks of the aromatic polymer, ultimate components, surface porosity, and so on [13]. With the widespread application of hydrochar, it is essential to develop efficient hydrothermal carbonization processes.

A substantial quantity of agricultural waste is generated annually, with rice straws being among the primary sources. These rice straws are commonly utilized for fuel production and as animal feed, which, unfortunately, can lead to secondary pollution [14,15]. Rice straw is classified as a lignocellulosic material, containing approximately 32–47% cellulose, 19–31.6% hemicellulose, 11–24% lignin, and 7–20% silica [14]. Due to its loose structure, rice straw is well-suited for hydrochar production. Researchers have previously explored the production of rice straw hydrochars using conventional heating methods [16,17]. Comparative analyses of their physical and chemical properties have been conducted in relation to rice straw pyrochars. These investigations have revealed that hydrochars exhibit a higher presence of oxygen-containing functional groups, which enhances their ability to adsorb pollutants [18]. Moreover, studies have demonstrated the effectiveness of rice straw hydrochars as adsorbents for removing metals [19].

Doping using heteroatoms can effectively alter the surface chemical properties of materials [20]. Chemical doping represents a viable strategy for the fabrication of novel biochars with enhanced pollutant adsorption capabilities. N-modified biochar can enhance the adsorption performance which is ascribed to introduce more functional groups [21]. The co-doping of nitrogen and phosphorus in biochar enhances its adsorption capacity for Pb^2+^ from various water bodies [22], which possible due to the surface complexation between functional groups and metal.

In this study, a toilet-sewage-based hydrothermal carbonation process was developed and the as-prepared hydrochar was used adsorbent in metal wastewater treatment. A significant increase in the hydrochar yield from raw straw was obtained when toilet sewage was used as solvent in the hydrothermal carbonization process. The as-prepared hydrochar materials were characterized using XRD, SEM-EDS, FTIR, XPS, and TEM. The kinetic, isotherm, and pH adsorption experiments explore the adsorption capacity performance of Cd^2+^ and Zn^2+^ using hydrochar. This finding would provide a simple process for the synthesis of functional carbon from raw biomass as an efficient absorbent for the metal wastewater with a low cost.

## 2. Materials and Methods

### 2.1. Materials

Analytical-grade KH_2_PO_4_, Urea, ethanol, metal salt (Zn(NO_3_)_2_·6H_2_O, and Cd(NO_3_)_2_·4H_2_O were purchased from Macklin Biochemical Co., Ltd. (Shanghai, China). Rice straw was obtained from Dali city, Yunnan province (China); this was washed with water and dried at 60 °C for 12 h. The metal (Cd^2+^, Zn^2+^) solutions were prepared using dissolving metal salt in ultrapure water (>18.25 MΩ). All the reagents were utilized as received, without treatment.

### 2.2. Synthesis and Characterization of Hydrochar

In a typical run, rice straw, urea (N), KH_2_PO_4_ (P), and ZrO_2_ balls (diameter 6.0 mm) were mixed using a ball-milling treatment (PM-100, RETSCH, Haan, Germany). After 10 min, the resulting powder was donated as R, or RNP, where R is the abbreviation of rice straw after the ball-milling treatment. For the hydrothermal synthesis of each modified hydrochar (depicted as RHC or RHCNP), 6 g of the ball-milling powder was added to 30 mL of water. Each sample was sonicated for 0.5 h, then loaded to an autoclave reactor (150 mL) and heated at 190 °C for 18 h. The obtained powder was washed with ethanol/water a couple of times. After being dried at 60 °C, the obtained powder was ground. The toilet-sewage-based hydrochar (depicted as RHC@FU and RHC@AFU) was prepared using hydrothermal ball-milling rice straw in FU or AFU; the addition amount was 0.01 mol N. The yields of hydrochar were calculated using the following equation:

(1) $$x=\frac{m}{M}$$

where *m* (g) is the mass of hydrochar and *M* (g) is the mass of rice straw dry weight.

Scanning electron microscopy (SEM) was used to analysis the microstructure of the as-prepared hydrochar. Energy-dispersive spectroscopy (EDS) of hydrcoahr was performed using the Octane Elect Super system from EDAX (Mahwah, NJ, USA). The pH drift method was used to analysis the point of zero charge of the as-prepared hydrochar adsorbent. The crystal structure of the hydrochar was characterized using X-ray diffraction (XRD) (Rigaku Ultima IV, Tokyo, Japan). The surface elements of the fresh and used hydrochar were determined using X-ray photoelectron spectroscopy (XPS) (Thermo Fisher, Waltham, MA, USA). The functional groups of the as-prepared hydrochar adsorbent were determined using Fourier-transform infrared (FTIR) spectroscopy (Thermo Fisher, Waltham, MA, USA). The specific surface areas of the as-prepared adsorbents were detected using nitrogen sorption on the surface area and a porosity analyzer (Micromeritics ASAP2460, Atlanta, USA), and were calculated using the Brunauer–Emmett–Teller method.

### 2.3. Adsorption Experiment

The bath adsorption experiments of the metal (Cd^2+^ or Zn^2+^) on the as-prepared hydrochar were carried out in single and binary-metal systems in a 50 mL centrifuge tube, where 20 mL of a solution with varying metal concentrations was used. The solution underwent agitation within a water bath shaker set at 25 °C and 160 rpm. Following a specified duration, the samples were subjected to centrifugation, and the metal concentration in the solution was quantified using inductively coupled plasma mass spectrometry (ICP-MS 7900, Agilent Technologies, Santa Clara, CA, USA). All results were replicated at least three times, and the reproducibility of the adsorption capacities were within a 3% standard deviation. The adsorptions of Cd^2+^ or Zn^2+^ were determined, utilizing the following formula:

(2) $$q_{t}=\frac{\left(C_{0}-C_{t}\right)\times V}{m}$$

where *q_t_* (mg g^−1^) is the adsorbing capacity of Cd^2+^ or Zn^2+^ at time *t*; *C_0_* (mg L^−1^) represents the initial concentration of Cd^2+^ or Zn^2+^; *C_t_* (mg L^−1^) stands for the concentration of Cd^2+^ or Zn^2+^ at time *t*; *V* (L) indicates the volume of the solution, and *m* (g) represents the mass of the hydrochar. 

Pseudo-first-order (PFO) and pseudo-second-order (PSO) models are widely employed in the fitting of adsorption data for N-doped biochars [23]. PFO and PSO were employed to elucidate the kinetics of the solution system, with PFO addressing a mononuclear adsorption process and PSO addressing a binuclear adsorption process [24].

PFO model:

(3) $$q_{t}=q_{e}(1-e^{-k_{1}t})$$

PSO model:

(4) $$q_{t}=\frac{k_{2}{q_{e}}^{2}t}{1+k_{2}q_{e}t}$$

where *q_t_* (mg g^−1^) represents the quantity of Cd^2+^ or Zn^2+^ adsorbed at a given time *t*; *q_e_* (mg g^−1^) is the amount of adsorbed Cd^2+^ or Zn^2+^ at equilibrium; *k*_1_ (min^−1^) and *k*_2_ (g mg^−1^ min^−1^) are the kinetic rate constants corresponding to the PFO and PSO models. Estimation of these parameters was carried out through a nonlinear regression approach.

The adsorption isotherm elucidates the mathematical correlation between the equilibrium concentration of a solute or adsorbate on the surface of an adsorbent and the concentration of that solute within its solution [23]. Among these, Langmuir and Freundlich models are the most prevalent in explaining the removal of contaminants from wastewater using activated carbon [25]. The Langmuir model postulates a uniform monolayer adsorption of solutes on a homogeneous adsorbent surface with specific adsorption sites [26]. The Freundlich isotherm approach assumes multilayer adsorption on a heterogeneous adsorbent surface [26]. The Sips isotherm model is a combined form of the Langmuir and Freundlich models, and can be used for multilayer adsorption on heterogeneous sites. To quantitatively describe the adsorption isotherms, the Langmuir, Freundlich, and Sips models were employed [27].

Langmuir isothermal adsorption equation:

(5) $$q_{e}=\frac{K_{L}q_{max}C_{e}}{1+K_{L}C_{e}}$$

Freundlich isothermal adsorption equation:

(6) $$q_{e}=K_{F}C_{e}^{\frac{1}{n}}$$

Sips isothermal adsorption equation:

(7) $$q_{e}=\frac{K_{LF}q_{max}C_{e}}{1+K_{LF}C_{e}}$$

where *q_e_* (mg g^−1^) signifies the quantity of Cd^2+^ or Zn^2+^ adsorbed at equilibrium, where *K_L_* represents the Langmuir constant associated with the adsorption energy. Additionally, *q_max_* (mg g^−1^) denotes the maximum adsorption capacity, *Ce* (mg L^−1^) represents the concentration of Cd^2+^ or Zn^2+^ at equilibrium, and *K_F_* and *n* are Freundlich isotherm constants linked to adsorption capacity and adsorption intensity, respectively. *K_LF_* represents the Sips constant associated with the adsorption energy.

The analysis of the rate-controlling step and the diffusion mechanism of metal adsorption by the prepared hydrochar involved the application of both the liquid-film diffusion (LFD) and intraparticle diffusion (IPD) models [28].

The LFD model may be described as

(8) $$L_{n}\left(1-F\right)=-K_{fd}t$$

where *K_fd_* (min^−1^) represents the adsorption rate constant, and *F* is the fractional attainment at equilibrium, denoted as (*F* = *q_t_*/*q_e_*).

The IPD is represented by

(9) $$q_{t}=-K_{i}t^{0.5}\;+\;C$$

where *K_i_* (mg g^−1^ min^−0.5^) represents the intraparticle diffusion rate constant and *C* (mg g^−1^) is a constant associated with the boundary layer thickness.

Comprehensive information can be found in the Supplementary Materials in Section SA.

## 3. Results and Discussion

### 3.1. Characterization Results

**Figure 1:**
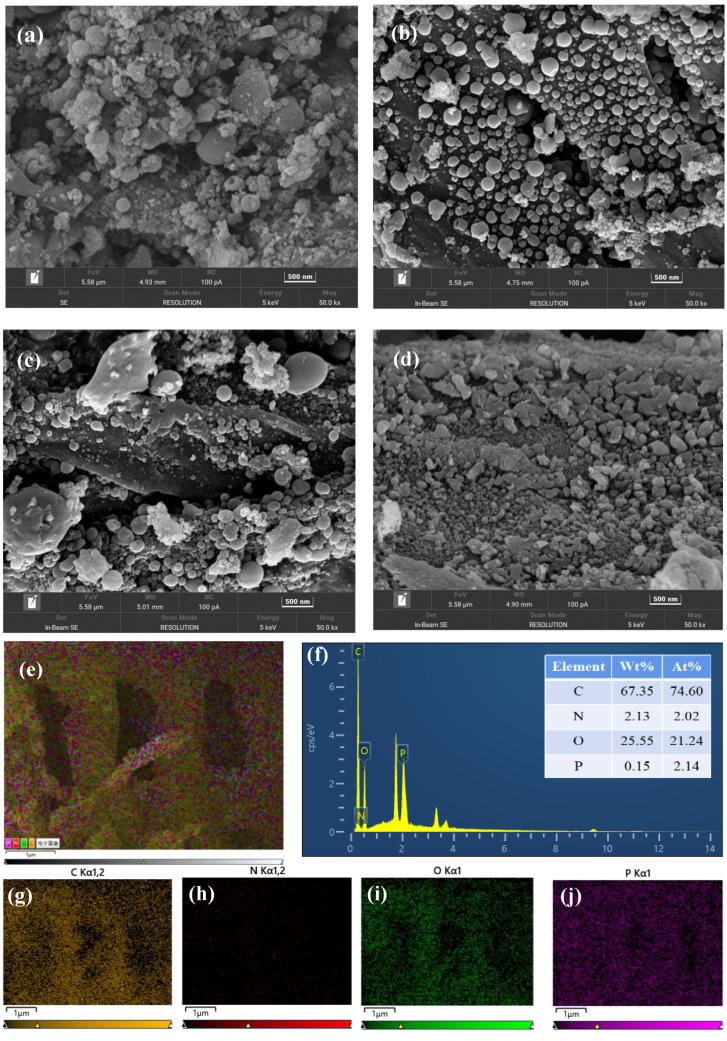
SEM images of (**a**) RHC, (**b**) RHCNP, (**c**) RHC@FU, and (**d**) RHC@AFU. (**e**) The elemental distribution images of RHCNP; (**f**) EDS and quantitatively element of RHCNP, (**g**) C, (**h**) N, (**i**) O, and (**j**) P distribution mapping of RHCNP.

An increased number of monodisperse carbonaceous spheres were identified in RHCNP, RHC@FU, and RHC@AFU, compared to RHC (Figure 1). The RHC, RHC@FU, and RHC@AFU samples exhibited rougher spheres (Figure 1). The RHCNP showed relatively uniform and smooth spheres in the range of 29–294 nm (Figure 1b). The EDS analysis results of RHCNP show homogeneous distribution of P and N on the hydrochar. The hydrochar yields of RHC, RHCNP, RHC@FU, and RHC@AFU were 62.4%, 63.6%, 96.7%, and 65.5%, respectively; these results suggest that a significant increase in the hydrochar yield was achieved with urine as solvent in the hydrothermal carbonization process. As shown in Table S1, the BET surface areas of the as-prepared hydrochar were lower than 10 m^2^ g^−1^.

**Figure 2:**
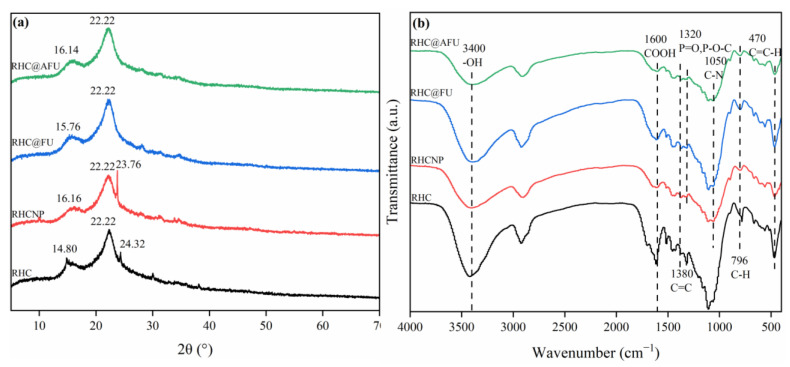
(**a**) X-ray diffraction patterns of raw-biomass-derived hydrochar adsorbent materials; (**b**) FT-IR spectra of raw-biomass-derived hydrochar adsorbent materials.

The XRD analysis of the hydrochars revealed peaks at 2θ = 14.80° and 22.22°, which corresponded to the characteristic crystalline structure of cellulose, specifically the (100) and (002) planes, respectively (Figure 2a) [10]. Notably, the cellulose peak notably decreased upon the addition of N/P, particularly at 16.1°. This reduction in the intensity of the cellulose peak suggests an increase in the degree of carbonization [29]. Consequently, it can be inferred that the raw biomass materials, namely RHCNP, RHC@FU, and RHC@AFU, underwent more extensive carbonization compared to RHC. Compared to RHC, XRD analysis of the modified hydrochar samples (RHCNP, RHC@FU, and RHC@AFU) showed a shift in the diffraction peaks of amorphous carbon. It is suggested that the introduction of phosphorus (P) and nitrogen (N) atoms into the carbon had occurred, and the N atoms made the interlayer space narrower. The peak shifts from 14.80° to 16.16°, 15.76°, and 16.14° indicate a change in the degree of lattice defects [29].

FT-IR analysis revealed that all the hydrochar samples exhibited peaks at approximately 1380 and 3400 cm^−1^, indicating the presence of C=C–H and COOH functional groups (Figure 2b) [30]. Compared to conventional hydrochar (RHC), the spectra of nitrogen-modified hydrochar (RHCNP), RHC@FU, and RHC@AFU exhibited different peaks at 1050 and 1320 cm^−1^, which were assigned to the C–N and P=O, P–O–C stretch vibration areas [31]. It is suggested that nitrogen and phosphorus were successfully incorporated into the modified hydrochar samples (RHCNP, RHC@FU, and RHC@AFU).

The chemical composition and state of carbon (C), nitrogen (N), and oxygen (O) in RHC, RHCNP, RHC@FU, and RHC@AFU were investigated using XPS analysis (Figure S1 and Table S1). The incorporation of N and P into the carbon framework was evident from the changes in the O 1 s spectra, which showed a reduction in C–O and the formation of C–N bonds. The N 1 s spectrum was divided into three peaks at 398.50 eV (Pyridinic–N), 399.80 eV (Pyrrolic–N), and 400.60 eV (Graphitic–N) [31]. The distribution of N in RHC was not clear, which was likely due to the low N content in the original feedstock. In contrast, the N 1 s peaks of RHCNP, RHC@FU, and RHC@AFU were prominent, suggesting the presence of a significant amount of N, with increases in Pyrrolic–N and Pyridinic–N.

### 3.2. Adsorption Capacity

**Figure 3:**
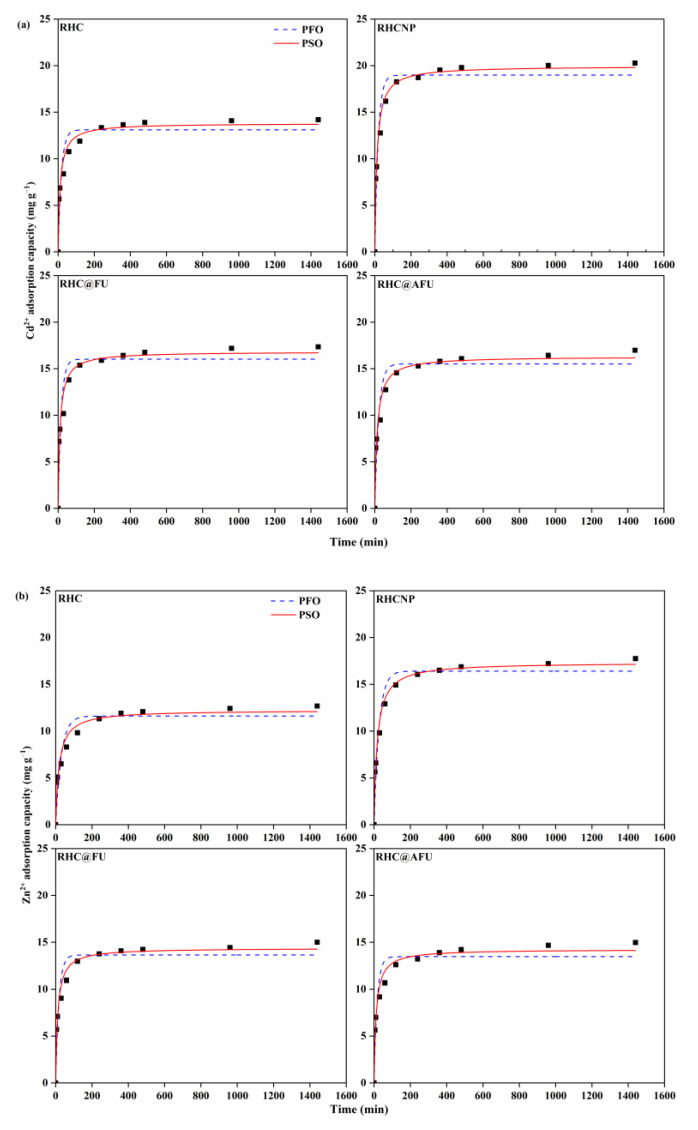

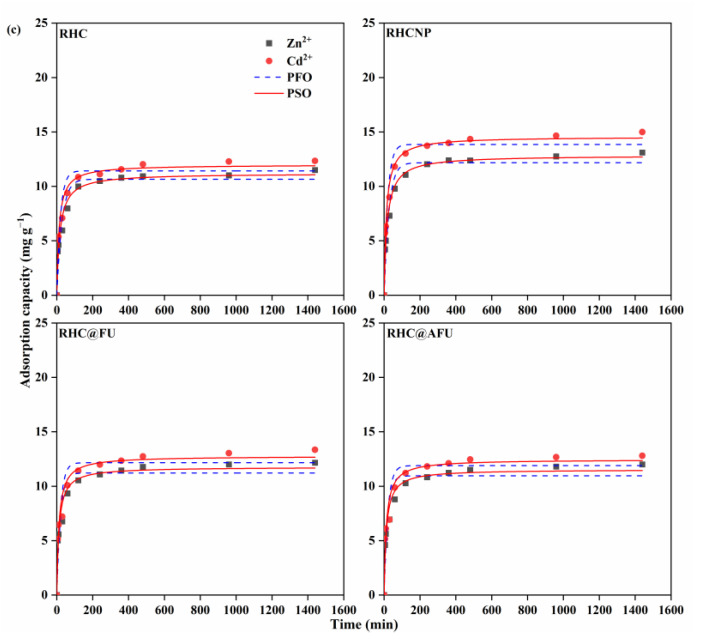
Adsorption capacity of metal ions (Cd^2+^ and Zn^2+^) on RHC, RHCNP, RHC@FU, and RHC@AFU in the single-metal system (**a**) Cd^2+^, (**b**) Zn^2+^ and (**c**) binary-metal system (solution concentration is 50 mg L^−1^, pH = 4.98, at 25 °C).

The adsorption capacity performance of prepared hydrochar (RHC, RHCNP, RHC@FU, and RHC@AFU) for metal ions (Cd^2+^ or Zn^2+^) in single or binary-metal systems, and their fittings with the pseudo-first-order model (PFO) and pseudo-second-order model (PSO) are presented in Figure 3a–c, the corresponding parameters of the models were given in Table 1. In the single metal (Cd^2+^) system, RHCNP, RHC@FU, and RHC@AFU exhibited a rapid increase in Cd^2+^ adsorption within 60 min, reaching equilibrium within 120 min, while RHC showed a slower adsorption rate, taking 240 min to reach equilibrium. It is suggested adsorption processes are followed with initial rapid adsorption and a prolonged slow rate. In single metal (Zn^2+^) system, RHCNP, RHC@FU, and RHC@AFU also showed a rapid increase in Zn^2+^ adsorption within 60 min, followed by a slow increase until reaching equilibrium at 120 min, while RHC showed a slower adsorption rate, taking 240 min to reach equilibrium. The adsorption rates of metal ions (Cd^2+^ and Zn^2+^) by hydrochar exhibited a rapid increase during the first 60 min, followed by a slower-paced increase until reaching equilibrium at around 240 min, in the binary system. The higher adsorption capacity of the as-prepared hydrochar to Cd^2+^ indicates that heavy metal ions (Cd^2+^) were susceptible to inner layer complexation.

The experimental adsorption capacity data of metal ions (Cd^2+^ and Zn^2+^) on all hydrochars in both the single system and the binary system were better fitted to the PSO model, which provides higher correlation coefficient values than that given by the PFO model (Table 1). It is suggested that the adsorption of metal ions (Cd^2+^ and Zn^2+^) onto the as-prepared hydrochar is a binuclear adsorption process. The adsorption capacity performance of different hydrochars for the metal ions (Cd^2+^ and Zn^2+^) followed a decreasing order of RHCNP > RHC@FU > RHC@AFU > RHC, in the single-metal system. For the PSO model, the adsorption capacity of RHCNP for Cd^2+^ was 19.96 mg g^−1^ at the equilibrium time, which is higher than RHC, RHC@FU, and RHC@AFU, respectively. The adsorption capacity of RHCNP for Zn^2+^ at the equilibrium time was greater than that of RHC, RHC@FU, and RHC@AFU, with a rate of increase of 41.6%, 20.7%, and 21.8%, respectively. It is suggested that RHCNP may as a promising adsorbent for the separation of metal ions (Cd^2+^ and Zn^2+^) from aqueous solution. EDS analysis indicated that RHCNP adsorbed 1.3% of Cd^2+^ and 0.5% of Zn^2+^ on the surface single-metal system, while adsorption with both ions resulted in 0.3% of Cd^2+^ and 0.2% of Zn^2+^, respectively, in the binary-metal system (Figure S2). Metal lattice streaks were observed in TEM images of RHCNP after adsorption. The EDS analysis revealed a decrease in N and an increase in P, suggesting their involvement in the adsorption process. For PSO model, the equilibrium adsorption capacity of as-prepared hydrochar for Cd^2+^ was 14.57 mg g^−1^ in the binary systems, which was higher than that of Zn^2+^ (12.85 mg g^−1^) (Table 1). This is possible due to the nitrogen and phosphorus doping, which enhances the surface electron distribution of hydrochar, providing more active adsorption sites [27].

**Figure 4:**
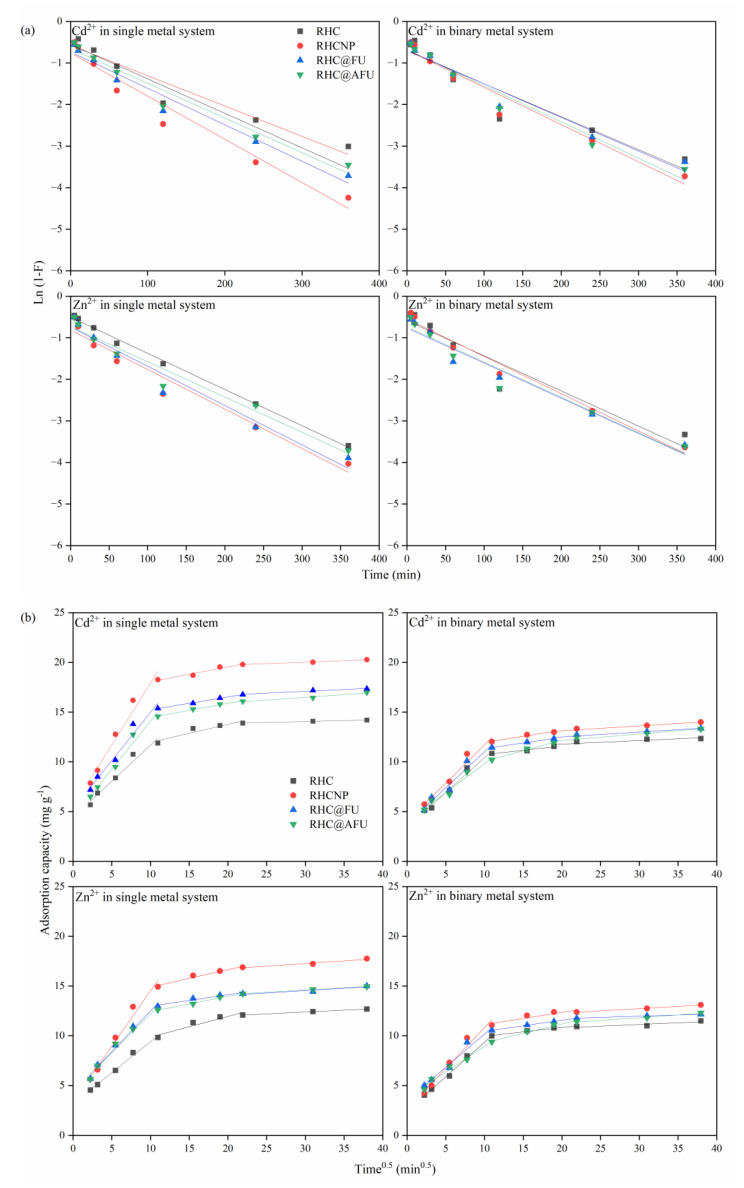
Fitted line of the (**a**) LFD and (**b**) IPD model for Cd^2+^ in the single system, Zn^2+^ in the single system, metal ions (Cd^2+^ or Zn^2+^) in the binary systems (Solution concentration is 50 mg L^−1^, pH = 4.98, at 25 °C).

The mechanisms of the adsorption processes were studied through the two-diffusion liquid-film diffusion (LFD) and intraparticle diffusion (IPF) models (Figure 4 and Table 2). For the experimental adsorption capacity of Cd^2+^, the LFD and IPD models fit well with relatively high R^2^, in both the single- and the binary-metal systems, indicating that both liquid-film diffusion and intraparticle diffusion played significant roles in the Cd^2+^ adsorption process. For the single Cd^2+^ adsorption process, the R^2^ of the IPD model on RHC was significantly smaller than that of other three modified hydrochar. It is suggested that, during the modification of hydrochar with N, P can improve the liquid-film diffusion. For the experimental adsorption capacity of Zn^2+^, the LFD and IPD models fit well with relatively high R^2^, in both the single- and binary-metal systems. It is suggested that intraparticle diffusion and liquid-film diffusion played a substantial role in the Zn^2+^ adsorption progress. The intra-granular diffusion was divided into three stages: surface diffusion, mesoporous diffusion, and microporous diffusion. The adsorption rate decreased gradually with Kd_1_ > Kd_2_ > Kd_3_.

### 3.3. Adsorption Isotherms

**Figure 5:**
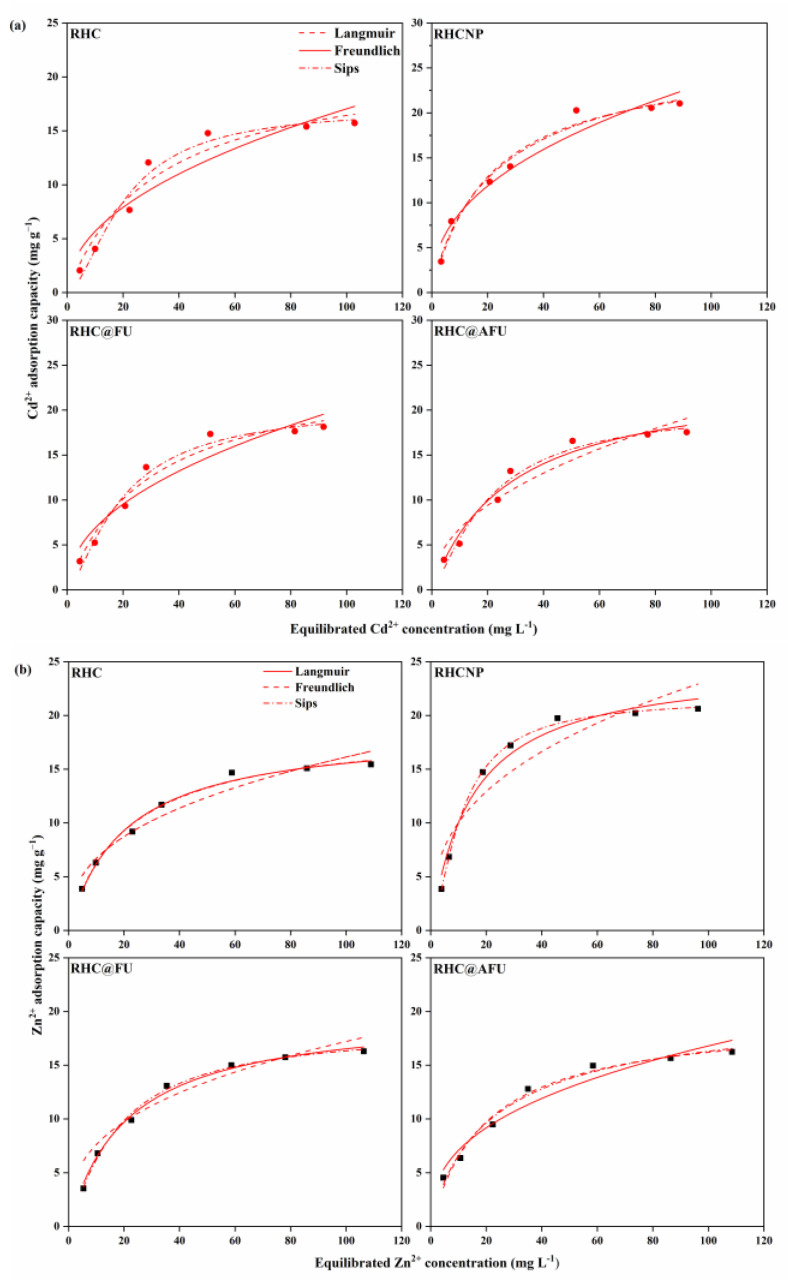

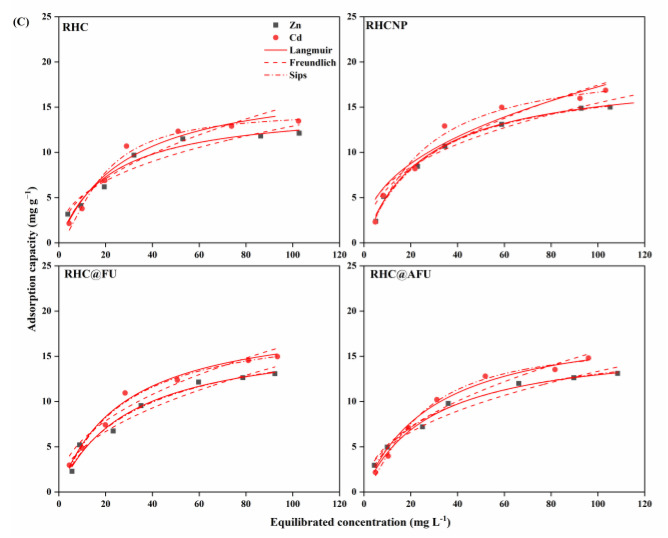
Adsorption isotherms of Cd^2+^ and Zn^2+^ on RHC, RHCNP, RHC@FU, and RHC@AFU in the single-metal systems. (**a**) Zn^2+^, (**b**) Cd^2+^, and (**c**) in the binary-metal system at 25 °C (solution concentration is 5–100 mg L^−1^, pH = 4.98).

Experimental adsorption isotherm data for heavy metal (Cd^2+^ or Zn^2+^) on the hydrochar and their fitting with the Langmuir, Freundlich, and Sips models were given in Figure 5 and Table 3. In both the single- and binary-metal systems, the experimental data will fit to the Sips models, which provided higher correlation coefficient values to that obtained by the Langmuir model and Freundlich model (Table 3). It is indicated that the adsorption process for both heavy metals (Cd^2+^ or Zn^2+^) could be simultaneously controlled by the Langmuir and Freundlich models, that is, by multiple processes. The Freundlich model parameter *k_F_* and n values was utilized to measure the affinity of hydrochar for metal ions and predict the thermodynamic adsorption capacity between hydrochar and metal ions during the sorption process, respectively. In this work, the 1/n value of all hydrochars is greater 1 for both single and binary metal adsorption systems (Table 3). It is suggested that the adsorption process of metal ions on hydrochars was favorable [32]. The parameter ‘n’ is a measure of adsorption intensity, quantifying the deviation from linearity in both single-metal systems and binary-metal systems [33]. An ‘n’ value of less than 1 indicates that the adsorption process is primarily chemical in nature [34].

In the single-metal systems, the as-prepared hydrochar RHCNP exhibited higher adsorption capacity for both metal ions. The maximum adsorption capacities of RHCNP, RHC@FU, and RHC@AFU for Cd^2+^ were 28.49, 20.48, and 20.52 mg g^−1^, respectively, which were higher than those for RHC (17.10 mg g^−1^). The maximum adsorption capacities of RHCNP, RHC@FU, and RHC@AFU for Zn^2+^ were 21.60, 18.53, and 20.87 mg g^−1^, respectively, which were higher than that of RHC (18.17 mg g^−1^), respectively. The adsorption capacities of metal ions (Cd^2+^ and Zn^2+^) on all hydrochars generally declined in the binary-metal system compared to in the single-metal system. This is possible due to the overlapping adsorption sites on the modified hydrochar [20]. The adsorption capacities of metal ions (Cd^2+^ and Zn^2+^) decreased in the following order: RHCNP>RHC@FU>RHC@AFU>RHC (Table 3). The Sips model revealed that the maximum adsorption capacities of RHCNP for the Cd^2+^ and Zn^2+^ were 21.63 and 18.82 mg g^−1^, respectively; these are higher than the adsorption capacities of RHC. The maximum adsorption capacities of RHC@FU and RHC@AFU for Cd^2+^ were around 18.80~19.12 mg g^−1^), while for Zn^2+^ were 18.07~18.52 mg g^−1^. These results show that the *q_max_* of as-prepared hydrochar for the Cd^2+^ was 2.8%~14.9% higher than that of Zn^2+^. The as-prepared hydrochars exhibited a higher adsorption capacity for Cd^2+^ than Zn^2+^. This is possible because Cd^2+^ has a larger ionic radius and higher relative atomic mass than Zn^2+^. The as-prepared N/P-doped rice straw hydrochars have comparable adsorption capacity performance to another adsorbent (Table 4) [6,21,35,36,37].

The surface competition between Cd^2+^ and Zn^2+^ and the impact of competitive ions on the adsorption capacity was analyzed and summarized in Table S2. The values of α were selectivity coefficients; it was indicated that the adsorption selectivity of the as-prepared hydrochar for the target metal is higher to that of competitive metal ions. Compared to the RHC, the Kd(Zn)and Kd(Cd) values for urine-modified hydrochar (RHC@FU and RHC@AFU) and N, P co-doping hydrochar (RHCNP) increased (Table S2). The α(Cd/Zn) values of urine-modified hydrochar (RHC@FU and RHC@AFU) and N/P doping hydrochar (RHCNP) were considerably greater than that for α(Zn/Cd). These results suggest that, after nitrogen and phosphorus activation or replacement of water by urine solution in the hydrothermal process, the hydrochar had a greater affect for heavy metal ions (Cd^2+^) than for Zn^2+^.

### 3.4. Effect of Solution pH

**Figure 6:**
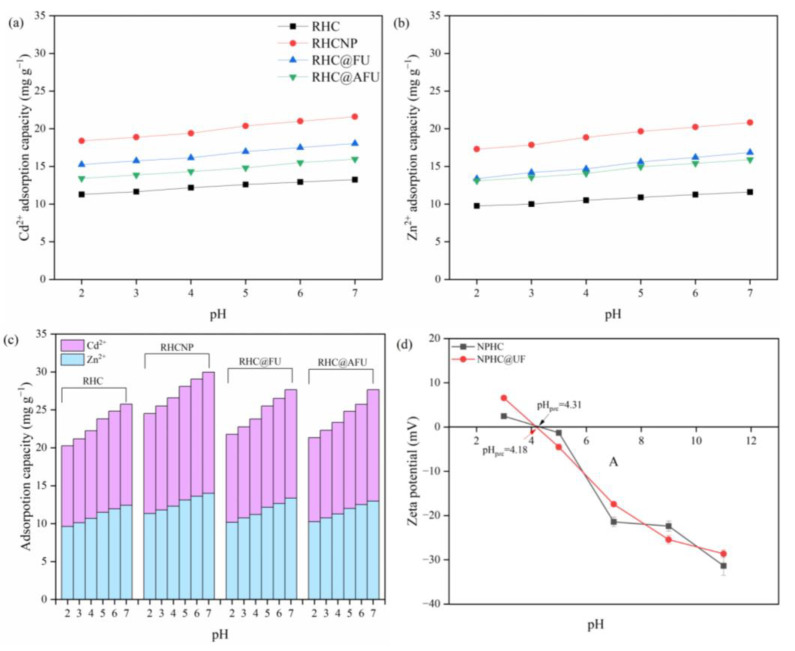
Effects of pH on the adsorption capacity of hydrochar for metal ions (Cd^2+^ and Zn^2+^) in the (**a**) single Cd^2+^ system, (**b**) single Zn^2+^ system, (**c**) binary-metal system, and (**d**) H_pzc_ of RHCNP and RHC@FU.

The effect of initial solution pH on the heavy metal adsorption capacities for the single- and binary-metal systems were studied (Figure 6). The initial pH suspension of the used materials in pure water was 4.98. Generally, the adsorption capacity of Cd^2+^ and Zn^2+^ increased with increasing pH from 2 to 7 in both single- and binary-metal systems (Figure 6). In the single system, the adsorption capacity of RHCNP for Cd^2+^ increased by 17.4% in the single system, while the adsorption capacity of RHCNP for Zn^2+^ increased by 20.4%. In the binary-metal system, the adsorption capacities of RHCNP for Cd^2+^ or Zn^2+^ increased by 21.1% and 23.6%, respectively. This is possible because the group electronegativity (e.g., COOH) plays significant role in the adsorption of metal [38]. The protons would compete for active sites on the hydrochar, which restricts the interaction between heavy metal ions and the absorbent at low pH system. When the pH increased from 4 to 5, the adsorption capacities of RHCNP and HC@FU significantly increased; this could be attributed to the pH_pzc_ of these two adsorbents, which falls within this range (Figure 6d). It is indicated that electrostatic interaction is a crucial mechanism for metal ions (Cd^2+^ and Zn^2+^) adsorption on the as-prepared hydrochar. Compared with Cd^2+^, the initial pH of the solution has a great affect on the adsorption capacity for Zn^2+^. The pH of both single- and binary-metal systems shows an initial increase followed by a subsequent decrease after adsorption (Figure S3). Initially, the basic functional groups (e.g., –NH_2_) on the hydrochar form a hydrogen bond with metal ions and hydrogen ions in the solution; this lowers the concentration of hydrogen ions in the solution. As the initial pH of the solution gradually increases, the deprotonation of adsorption sites ensues, leading to a decrease in the pH values. The decrease in the pH after adsorption suggested that the ion exchange occurs between metal ions and acidic functional groups on the hydrochar [27].

**Figure 7:**
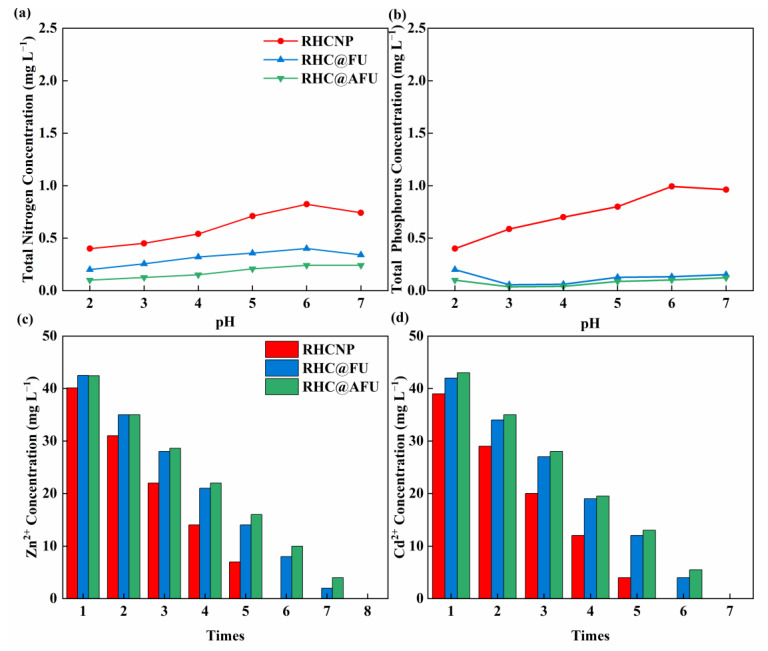
(**a**) N and P (**b**) leached from the adsorbent at different pH, (**c**) Zn^2+^, and (**d**) Cd^2+^ concentration after multiple adsorptions.

In order to assess the stability of the materials, a series of experiments were conducted to measure the N/P concentrations released from the materials at different pH values. As shown in Figure 7a,b, the released N/P concentrations into the solution initially increased and then decreased, as the pH increased. When the pH was 6, the maximum released N concentrations into the solutions for RHCNP, RHC@FU, and RHC@AFU were 0.823, 0.402, and 0.242 mg L^−1^, respectively. The maximum released P concentrations into the solutions for RHCNP, RHC@FU, and RHC@AFU were 0.993, 0.132, and 0.102 mg L^−1^, respectively. The as-prepared hydrochar was stable under a pH between 2 and 7. The results of multiple repeated experiments demonstrate that the as-prepared hydrochars are capable of complete removal of metal from solution after 5–7 rounds (Figure 7c,d).

### 3.5. Mechanisms

**Figure 8:**
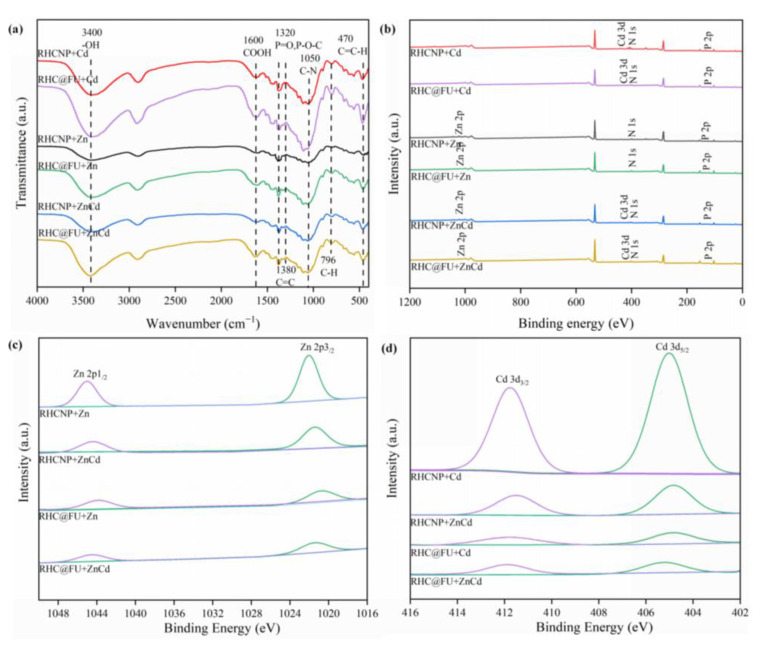
Analyses of RHCNP and RHC@FU after adsorption with Cd^2+^ and Zn^2+^ using (**a**) Fourier transform infrared (FTIR); (**b**) X-ray photoelectron spectroscopy (XPS); (**c**) Cd_3d_; (**d**) Zn_2p_.

**Figure 9:**
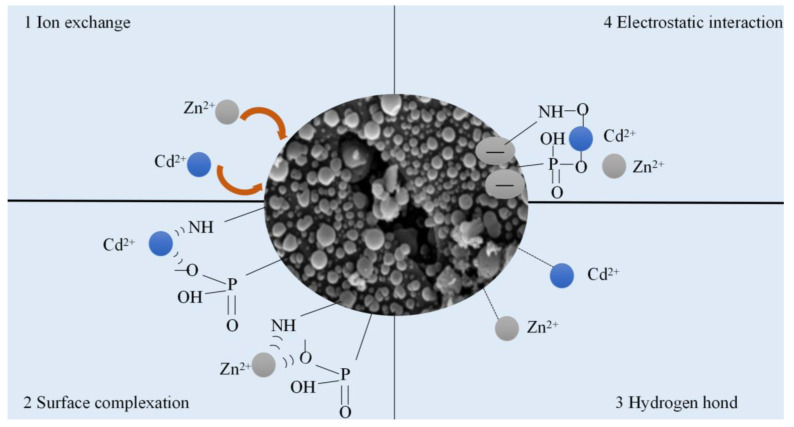
Proposed synthesis mechanism of N/P-doped rice straw based hydrochar.

To further illuminate the interactions between the modified rice straw hydrochar and Cd^2+^ and Zn^2+^, FT-IR (Figure 8a) and XPS measurements (Figure 8b–d) were carried out on fresh and spent adsorbents. The intensity of N 1s of RHCNP and RHC@FU was significantly changed after the adsorption experiment. It is suggested that N was involved in the adsorption process (Figure 8b). The Cd _3d_ spectra of the used adsorbent (RHCNP and RHC@FU) were composed of Cd 3d_3/2_ (412.4 eV) and Cd 3d_5/2_ (405.6 eV) (Figure 8d). These results indicate that the hydroxyl group might participate in the Cd^2+^ adsorption by forming Cd–O bonds [21]. The Zn 2p spectra of Zn–O bonds comprised peaks at 1045.5 eV and1022.4 eV, which were assigned to Zn 2p_1/2_ and Zn 2p_3/2_. The characteristic peaks at 3400 cm^−1^ (–OH) and 1591 cm^−1^ (–COOH) of the used adsorbent were weaker than that of fresh adsorbent. This is possible because Cd^2+^ and Zn^2+^ react with the oxygen-containing group on the hydrochar through forming complexes. The results of this study suggest that complexation reactions played a crucial role in the adsorption of Cd^2+^ and Zn^2+^ by RHCNP. Based on the above results, the adsorption mechanism of the enhanced metal ions (Cd^2+^ and Zn^2+^) removal using modified rice straw is illustrated in Figure 9, including electrostatic interactions, hydrogen bonding, and ion exchange.

### 3.6. Research Implications

**Figure 10:**
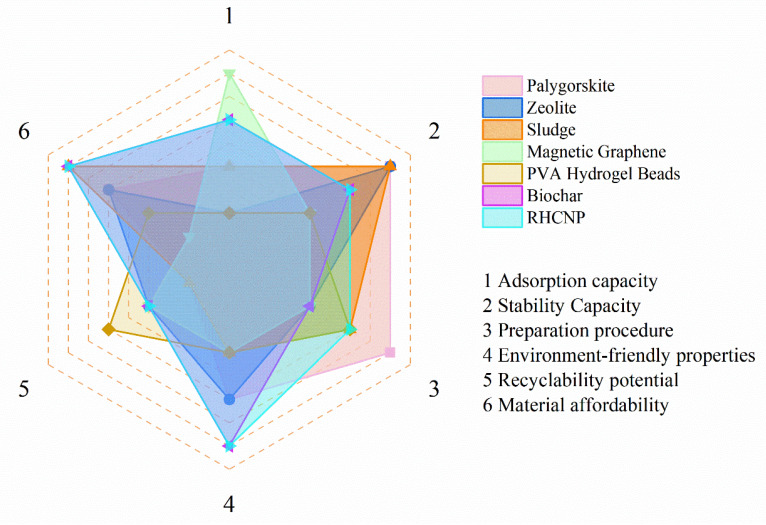
Radar map for comparison of comprehensive Cd^2+^ and Zn^2+^ adsorption performance compared with different adsorbents according to six criteria.

The adsorbent of the adsorptive removal of Cd^2+^ or Zn^2+^ from aqueous media depends on the adsorption capacity, stability capacity, preparation procedure, environmentally friendly properties, recyclability potential, and the affordability of the adsorbent. However, most studies in recent years have primarily focused on improving the heavy metal removal capacity of adsorbents, neglecting the eco-friendliness of their properties. Pursuing high sorption capacity alone is unsustainable and inadequate for designing satisfactory heavy metal adsorbents, because sorption performance depends on several factors. In this study, RHCNP, RHC@FU, and RHC@AFU exhibited commendably high sorption capacity and eco-friendliness. However, the relatively lower recollection potential of powder adsorbents due to their small mass compared with polymer matrices was observed. Thus, we developed a novel evaluation system that utilizes a radar map to evaluate heavy metal adsorption capacity, material stability, cost, environmental friendliness, recyclability potential, etc. Represented by different colored lines, a comparison between the Cd^2+^ or Zn^2+^ separation performances of other adsorbents is presented. All the adsorbents showed some disadvantages for all five properties. RHCNP, RHC@FU, and RHC@AFU demonstrated lower sorption recyclability potential than the seven other adsorbents (Figure 10 and Table S3). However, their capacity, preparation simplicity, and environmental friendliness were far superior. Overall, RHCNP was identified as a promising Cd^2+^ or Zn^2+^ adsorbent compared to common adsorbents.

The maximum adsorption capacities of all the adsorbents (1–7) for cadmium were 10.4, 1.8, 76.1, 128.2, 98.1, 10.2, and 26.3 mg g^−1^, respectively. The maximum adsorption capacities of the same adsorbents for Zn^2+^ ions were 17.1, 1.1, 5.3, 62.9, 40.8, 12.1, and 24.9 mg g^−1^, respectively. The matrix materials of these adsorbents included attapulgite, zeolite, sludge, graphene, polyvinyl alcohol, activated larch, and rice straw. Attapulgite and zeolite are typically priced at EUR 65–129.6 per ton, while polyvinyl alcohol costs around EUR 2593 per ton [39]. Activated larch and rice straw are generally priced at EUR 25–80 per ton. Graphene is expensive, with an international market price of EUR 94 per gram. Sludge can be obtained free of charge from sewage treatment plants [40]. The preparation process for these adsorbents varies, requiring the addition of various reagents and equipment to ensure proper environmental conditions. In this process, waste reagents may be generated, resulting in potential environmental risks. Additionally, the physical and chemical properties of these adsorbents can affect the environment after application. For example, the palygorskite matrix itself has relatively little impact on the environment, but adsorbents modified with metals and reagents have the potential risk of metal release. Furthermore, powdered adsorbents are difficult to recover after application, while micron granular adsorbents can be recovered by simple filtration [41].

## 4. Conclusions

In this work, N- and P-co-doped hydrochars were prepared using the hydrothermal treatment of rice straw with water or urine as the hydrothermal solution. The N- and P-co-doped and urine-modified hydrochars enhanced the adsorption capacities toward Cd^2+^ and Zn^2+^ ions compared with non-modified hydrochar. In both single- and binary-metal systems, the adsorption equilibrium following the Sips models suggested that metal ions adsorption could be simultaneously controlled by multiple processes. The Cd^2+^ and Zn^2+^ removal ability of the modified hydrocar can be attributed to the hydrochar’s surface complexation, electrostatic interaction, hydrogen bond, and ion exchange. As a low-cost material (urine and rice straw), biomass-derived hydrochar can be regarded as an effective adsorbent for the treatment of Cd^2+^ and Zn^2+^.

## Figures and Tables

**Table 1:** Parameters of pseudo-first-order model (PFO) and pseudo-second-order model (PSO) for metal ions (Cd^2+^ and Zn^2+^) adsorption kinetics on RHC, RHCNP, RHC@FU, and RHC@AFU in the single-metal systems or binary-metal systems.

Metal Ion	Adsorbent		PFO			PSO	
		*q_e_* (mg g^−1^)	*K*_1_ (min^−1^)	R^2^	*q_e_* (mg g^−1^)	*K*_2_ (g mg^−1^ min^−1^)	R^2^
Cd^2+^	RHC	13.11	0.0553	0.899	13.82	0.0062	0.967
	RHCNP	18.99	0.0544	0.937	19.96	0.0043	0.984
	RHC@FU	16.02	0.0590	0.907	16.83	0.0056	0.969
	RHC@AFU	15.52	0.0466	0.912	16.31	0.0047	0.970
Zn^2+^	RHC	11.61	0.0321	0.888	12.24	0.0044	0.954
	RHCNP	16.41	0.0360	0.945	17.34	0.0039	0.985
	RHC@FU	13.63	0.0558	0.912	14.37	0.0060	0.975
	RHC@AFU	13.46	0.0578	0.907	14.24	0.0598	0.972
Cd^2+^ (Cd^2+^ + Zn^2+^)	RHC	11.42	0.0484	0.904	11.99	0.0067	0.964
	RHCNP	13.85	0.0494	0.934	14.57	0.0054	0.981
	RHC@FU	12.16	0.0502	0.894	12.77	0.0064	0.960
	RHC@AFU	11.89	0.0481	0.895	12.46	0.0065	0.959
Zn^2+^ (Cd^2+^ + Zn^2+^)	RHC	10.66	0.0342	0.919	11.19	0.0053	0.937
	RHCNP	12.17	0.0374	0.946	12.85	0.0046	0.985
	RHC@FU	11.22	0.0496	0.897	11.77	0.0070	0.961
	RHC@AFU	10.96	0.0515	0.903	11.54	0.0070	0.969

**Table 2:** Liquid-film diffusion (LFD) and intraparticle diffusion (IPF) parameters for Cd^2+^ and Zn^2+^.

Metal Ion	Adsorbent		LFD		IPD								
		*K_fd_*	*C*	R^2^	*K*_*i*1_	*C*	*R* ^2^	*K*_*i*2_	*C*	R^2^	*K*_*i*3_	*C*	*R* ^2^
		(min^−1^)	(mg L^−1^)		(mg g^−1^ min^−0.5^)	(mg L^−1^)		(mg g^−1^ min^−0.5^)	(mg L^−1^)		(mg g^−1^ min^−0.5^)	(mg L^−1^)	
Cd^2+^	RHC	0.007	−0.58	0.938	0.72	4.44	0.967	0.18	10.17	0.888	0.02	13.49	0.993
	RHCNP	0.010	−0.76	0.955	1.24	5.52	0.971	0.15	16.58	0.965	0.03	19.14	0.990
	RHC@FU	0.009	−0.73	0.970	0.97	5.28	0.968	0.13	13.95	0.997	0.04	15.99	0.968
	RHC@AFU	0.008	−0.66	0.967	0.96	4.46	0.980	0.14	13.08	0.991	0.06	14.82	0.962
Zn^2+^	RHC	0.009	−0.50	0.997	0.62	3.17	0.993	0.21	7.78	0.918	0.04	11.26	1.000
	RHCNP	0.010	−0.81	0.961	1.11	3.39	0.976	0.18	13.12	0.967	0.05	15.66	0.957
	RHC@FU	0.009	−0.74	0.960	0.82	4.31	0.986	0.12	11.81	0.943	0.05	13.13	0.886
	RHC@AFU	0.008	−0.73	0.962	0.78	4.41	0.977	0.15	10.90	0.992	0.05	13.22	0.998
Cd^2+^ (Cd^2+^ + Zn^2+^)	RHC	0.008	−0.71	0.902	0.70	3.41	0.981	0.09	9.85	0.955	0.04	11.06	0.773
	RHCNP	0.009	−0.69	0.958	0.77	4.03	0.969	0.12	10.72	0.964	0.05	12.17	0.960
	RHC@FU	0.008	−0.69	0.960	0.72	3.78	0.961	0.11	10.19	0.941	0.05	11.53	0.968
	RHC@AFU	0.009	−0.68	0.964	0.58	4.00	0.968	0.21	7.91	0.989	0.07	10.81	0.936
Zn^2+^ (Cd^2+^ + Zn^2+^)	RHC	0.008	−0.59	0.927	0.70	2.40	0.995	0.09	9.08	0.973	0.03	10.10	0.803
	RHCNP	0.009	−0.55	0.981	0.82	2.60	0.969	0.12	9.89	0.864	0.05	11.38	0.996
	RHC@FU	0.008	−0.76	0.939	0.67	3.50	0.971	0.11	9.34	0.999	0.02	11.23	0.997
	RHC@AFU	0.009	−0.73	0.954	0.51	3.78	0.977	0.19	7.40	0.976	0.06	10.15	0.980

**Table 3:** Langmuir and Freundlich parameters for the Cd^2+^ and Zn^2+^ adsorption isotherms on RHC, RHCNP, RHC@FU, and RHC@AFU in the single and binary systems.

Metal Ion	Adsorbent	Langmuir			Freundlich			Spis			
		*q_max_* (mg g^−1^)	*K_L_* (L mg^−1^)	R^2^	n	*K_F_* (mg g^−1^)	*R* ^2^	*q_max_* (mg g^−1^)	n	*K_LF_* (L mg^−1^)	*R* ^2^
Cd^2+^	RHC	21.67	0.031	0.951	0.529	2.094	0.885	17.10	1.674	0.006	0.976
	RHCNP	26.30	0.048	0.931	0.301	2.355	0.954	28.49	0.056	0.893	0.980
	RHC@FU	24.75	0.035	0.969	0.446	2.124	0.912	20.48	1.437	0.014	0.981
	RHC@AFU	23.98	0.035	0.972	0.419	1.136	0.922	20.52	0.019	1.318	0.980
Zn^2+^	RHC	18.71	0.049	0.989	0.359	2.619	0.949	18.17	0.053	0.957	0.989
	RHCNP	24.86	0.068	0.979	0.231	2.737	0.873	21.60	0.029	1.479	0.999
	RHC@FU	20.06	0.047	0.993	0.362	2.512	0.963	18.53	0.034	1.170	0.995
	RHC@AFU	19.44	0.050	0.981	0.336	2.663	0.944	20.87	0.060	0.886	0.986
Cd^2+^ (Cd^2+^ + Zn^2+^)	RHC	17.89	0.035	0.967	0.588	2.101	0.909	15.66	0.018	1.317	0.974
	RHCNP	21.73	0.034	0.980	0.483	2.162	0.936	21.63	0.024	1.181	0.983
	RHC@FU	19.47	0.036	0.984	0.627	1.310	0.945	19.12	0.038	1.006	0.986
	RHC@AFU	19.17	0.033	0.971	0.579	2.095	0.911	18.80	0.018	1.217	0.993
Zn^2+^ (Cd^2+^ + Zn^2+^)	RHC	15.04	0.047	0.964	0.475	2.539	0.925	15.23	0.049	0.978	0.964
	RHCNP	19.10	0.036	0.966	0.448	2.384	0.966	18.82	0.035	1.022	0.994
	RHC@FU	17.44	0.034	0.973	0.622	2.104	0.956	18.52	0.038	0.038	0.974
	RHC@AFU	16.64	0.035	0.962	0.556	2.298	0.958	18.07	0.053	0.817	0.990

**Table 4:** Summary of various biochars adsorbents for Cd(II) and Zn(II) ions uptake.

Adsorbents	Metal Ion	Initial Concentration (mg g^−1^)	Adsorbent Dose (g L^−1^)	Equilibrium Time (h)	*q_max_* (mg g^−1^)	Reference
Agricultural wastes biochars	Cd^2+^	0–100	1	-	6.28	[35]
KMnO_4_ and hematite modified biochar	Cd^2+^	0–100	2.5	2	34.25	[36]
N-doping biochar	Cd^2+^	0–150	1	8	8.72	[21]
RHCNP	Cd^2+^	0–100	0.5	1	26.30	This work
Cement-biochar composite	Zn^2+^	10	0.5	4	19.0	[6]
KOH modified biochar	Zn^2+^	0–100	3	8	97.68	[37]
KMnO_4_ and hematite modified biochar	Zn^2+^	0–100	2.5	2	17.92	[36]
RHCNP	Zn^2+^	0–100	0.5	1	24.86	This work

## Data Availability

Data are contained within the article and Supplementary Materials.

## Supplementary Materials

The following supporting information can be downloaded at: https://www.mdpi.com/article/10.3390/polym15234548/s1. Figure S1. XPS spectra of hydrochar adsorbent materials; Figure S2. (a,b) EDS spectra of RHCNP after adsorption with Cd^2+^; (d,e) RHCNP after adsorption with Zn^2+^; (g,h) RHCNP after adsorption with Cd^2+^ and Zn^2+^; TEM images of (c) RHCNP after adsorption with Cd^2+^; (f) RHCNP after adsorption with Zn^2+^; (i) RHCNP after adsorption with Cd^2+^ and Zn^2+^; Figure S3. The pH of the solution before and after adsorption ((a) single Cd2+ system, (b) single Zn^2+^ system, and (c) binary metal system); Table S1. Isothermals analysis and X-ray photoelectron spectroscopy of as-prepared adsorbents; Table S2. Distribution coefficient (Kd) and selectivity coefficient (α) under competitive adsorption conditions; Table S3. Comparison of comprehensive Cd^2+^ and Zn^2+^ adsorption performance by different adsorbents. Refs. [24,27,42,43,44,45,46,47,48,49,50,51] are cited in the Supplementary Materials.
