# Supplementary material for: Hydrothermal Ammonia Carbonization of Rice Straw for Hydrochar to Separate Cd(II) and Zn(II) Ions from Aqueous Solution
Source: Polymers (Basel). 2023 Nov 27;15(23):4548. doi: 10.3390/polym15234548 (PMC10708519; doi:10.3390/polym15234548)
Supplement: Supplementary file 1 [file polymers-15-04548-s001.zip › polymers-2699929-supplementary.pdf]

polymers

MDPI

Supplementary Material

Hydrothermal Ammonia Carbonization of Rice Straw for Hydrochar to Separate Cd(II) and Zn(II) Ions from Aqueous Solution

Jiarui Wang [1,2,†], Xiaocheng Wei [1,2,†], Hao Kong [1,2], Xiangqun Zheng [1,2,3,*] and Haixin Guo [1,2,*]

[1] Agro-Environmental Protection Institute, Ministry of Agriculture and Rural Affairs, No. 31 Fukang Road, Nankai District, Tianjin 300191, China; wang_jiar@163.com (J.W.); 13752221539@163.com (X.W.); hnkonghao@163.com (H.K.)
[2] Key Laboratory for Rural Toilet and Sewage Treatment Technology, Ministry of Agriculture and Rural Affairs, Tianjin 300191, China
[3] Institute of Environment and Sustainable Development in Agriculture, CAAS, Beijing 100081, China
* Correspondence: zhengxiangqun@126.com (X.Z.); haixin_g@126.com (H.G.)
† These authors contributed equally to this work.

Section A. Adsorption experiments

Bath adsorption experiments of the metal (Cd^{2+} or Zn^{2+}) on the hydrochar were performed using single- and binary-metal systems. The kinetic adsorption experiments for the single-metal system were carried out using a 50 mL centrifuge tube containing 20 mL of 50 mg L^{-1} Cd^{2+} or Zn^{2+} solution, with 0.01 g each of RHC, RHCNP, RHC@FU, and RHC@AFU. The solution was agitated in a water bath shaker (25 °C, 160 rpm) and samples were collected at regular intervals (5, 10, 30, 60, 120, 240, 360, 480, 960, and 1440 min) in triplicate. Subsequently, the samples were centrifuged, and the supernatants were filtered through 0.45 μm nylon membrane filters. The Cd^{2+} or Zn^{2+} concentrations in the supernatants were determined using inductively coupled plasma mass spectrometry (ICP-MS; 7900, Agilent Technologies, USA). The adsorbed amounts of Cd^{2+} or Zn^{2+} were calculated using the following equation:

$$q_t = \frac{(C_0 - C_t) * V}{m} \quad (1)$$

where q_t (mg g^{-1}) is the adsorbing capacity of Cd^{2+} or Zn^{2+} by hydrochar at time *t*; C_0 (mg L^{-1}) is the initial Cd^{2+} or Zn^{2+} concentration; Ct (mg L^{-1}) is the Cd^{2+} or Zn^{2+} concentration at time *t*; *V* (L) is the volume of the solution; and *m* (g) is the mass of hydrochar.

Lagrange's pseudo-first-order (PFO) and pseudo-second-order (PSO) models were used to describe the kinetics of Cd^{2+} or Zn^{2+} adsorption by hydrochar. The PFO model was used to describe the kinetics of solid solution system based on a mononuclear adsorption process, while the PSO model was used to describe the kinetics of solid solution system based on a binuclear adsorption process [1].

PFO model:

$$q_t = q_e(1 - e^{-k_1 t}) \quad (2)$$

PSO model:

$$q_t = \frac{k_2 q_e^2 t}{1 + k_2 q_e t} \quad (3)$$

where q_t (mg g^{-1}) is the amount of adsorbed Cd^{2+} or Zn^{2+} at time t; q_e (mg g^{-1}) is the amount of adsorbed Cd^{2+} or Zn^{2+} at equilibrium, and k_1 (h^{-1}) and k_2 (g mg^{-1} h^{1}) are the kinetic rate constants of PFO and PSO models. The parameters were estimated using nonlinear regression.

The liquid-film diffusion (LFD) and intraparticle diffusion (IPD) models were used to analyze the rate-controlling step and the diffusion mechanism of Cd^{2+}/Zn^{2+} adsorption by RHC, RHCNP, RHC@FU, and RHC@AFU. The LFD model may be described as

$$L_n(1 - F) = -K_{fd} t \quad (4)$$

where K_{fd} (min^{-1}) is the adsorption rate constant and F is the fractional achievement at equilibrium ($F = q_t/q_e$).

The IPD is represented by

$$q_t = K_i t^{0.5} + C \quad (5)$$

where K_i (mg g^{-1} min$^{0.5}$) is the intraparticle diffusion rate constant and C (mg g^{-1}) is a constant related to the boundary layer thickness.

For the binary metal system, 20 mL of 50 mg L^{-1} Cd^{2+} and 20 mL of 50 mg L^{-1} Zn^{2+} were added to a 50 mL centrifuge tube containing 0.01 g hydrochar. Further steps were the same as that followed for the single metal system.

Isotherm studies on the single metal system were performed using 20 mL of Cd^{2+} (5-100 mg L^{-1}) or Zn^{2+} (5-100 mg L^{-1}) solutions with 0.01 g of RHC, RHCNP, RHC@FU, and RHC@AFU in a 50 mL centrifuge tube. The solution was agitated in a water bath shaker (at 25 °C, 160 rpm) for 24 h. For isotherm studies on binary metal system adsorption, 10 mL of Cd^{2+} (5-100 mg L^{-1}) and 10 mL of Zn^{2+} (5-100 mg L^{-1}) were added to a 50 mL centrifuge tube containing 0.01 g of RHC, RHCNP, RHC@FU, and RHC@AFU. The Langmuir, Freundlich, and Sips models were used to quantitatively describe the adsorption isotherms of Cd^{2+} and Zn^{2+}.

Langmuir isothermal adsorption equation：

$$q_e = \frac{K_L q_{max} C_e}{1+K_L C_e} \quad (6)$$

Freundlich isothermal adsorption equation：

$$q_e = K_F C_e^{\frac{1}{n}} \quad (7)$$

Sips isothermal adsorption equation：

$$q_e = \frac{K_{LF} q_{max} C_e}{1+K_{LF} C_e} \quad (8)$$

where q_e (mg g^{-1}) is the amount of adsorbed Cd^{2+} or Zn^{2+} at equilibrium; k_L is the Langmuir constant related to the energy of adsorption; q_{max} (mg g^{-1}) is the maximum adsorption capacity; C_e (mg L^{-1}) is the concentration of Cd^{2+} or Zn^{2+} at equilibrium; and k_F and n are Freundlich isotherm constants related to adsorption capacity and adsorption intensity, respectively; k_{LF} is the Langmuir constant related to the energy of adsorption.

Section B. Statistical analysis

The experimental data underwent one-way analysis of variance (ANOVA) using SPSS 25 software. The significant effects of various hydrochar applications were determined by identifying the least significant

differences value at $P \leq 0.05$. To analyze the adsorption selectivity of Cd^{2+} and Zn^{2+} by hydrochar, a distribution coefficient (K_d) was employed, which is defined as follows [2]:

$$K_d = \frac{V * (C_0 - C_e)}{m * C_e} \quad (9)$$

where C_0 and C_e (mg L^{-1}) are the concentrations of Cd^{2+} or Zn^{2+} at initial and equilibrium under the binary metal system, respectively; V(L) is the volume of the solution; and m (mg) is the mass of hydrochar.

The selectivity coefficient (α), the ratio of K_d values for the two metals in the binary metal system, was used to describe the competitive adsorption capacity of a particular metal ion [3]:

$$a = \frac{K_d(\mathrm{T})}{K_d(\mathrm{I})} \quad (10)$$

where K_d (T) is the K_d value of the targeted metal ion and K_d (I) is the K_d value of another metal ion in the binary metal system.

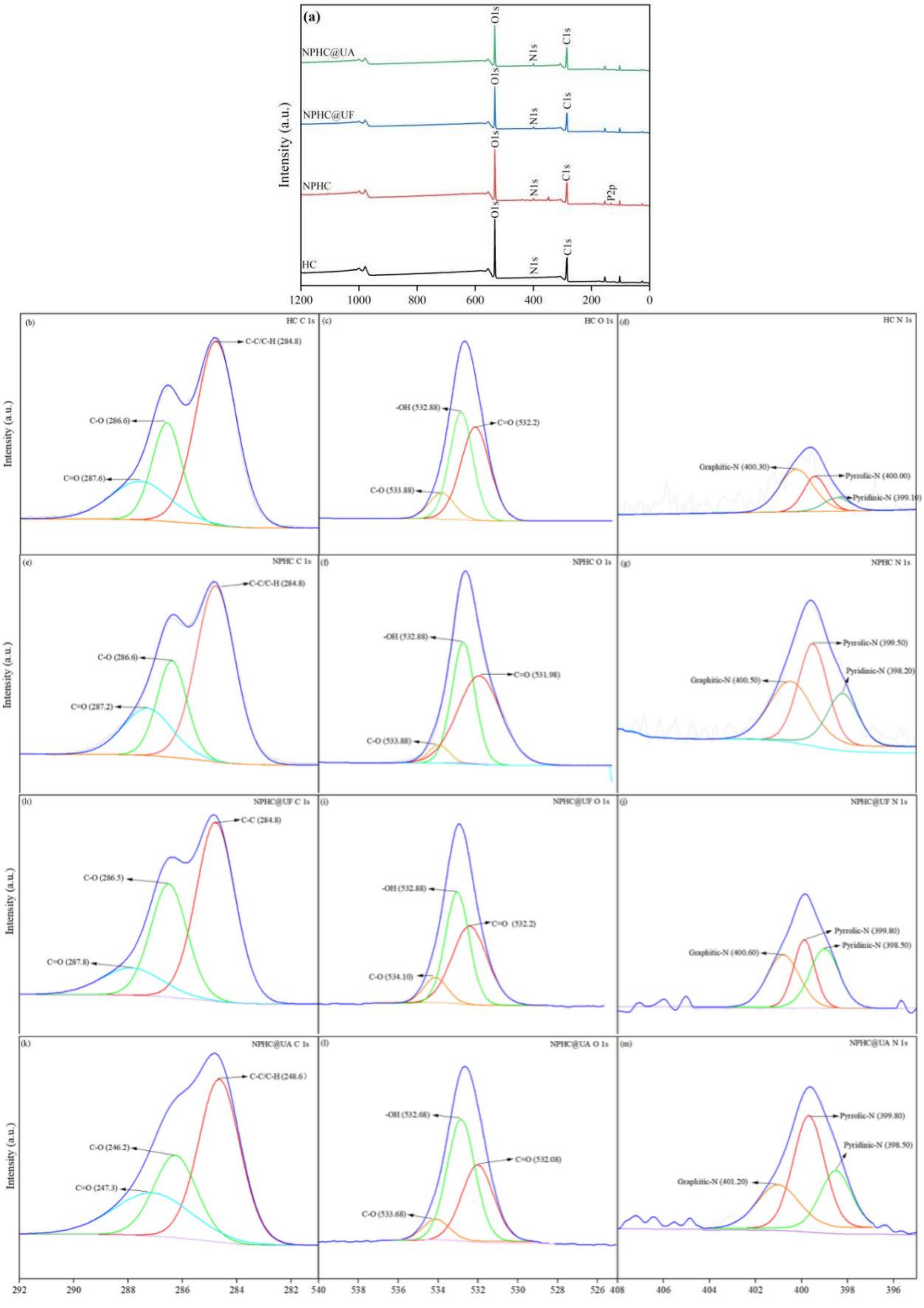

Figure S1. XPS spectra of hydrochar adsorbent materials.

Table S1. Isothermals analysis and X-ray photoelectron spectroscopy of as-prepared adsorbents.

Samples	S_{BET}	C 1_S			O 1_S			N 1_S		
	$m^2\ g^{-1}$	C−C/C−H	C−O	C=O	C=O	−OH	C−O	Pyridinic−N	Pyrrolic−N	Graphitic−N
RHC	2.1	58.49	23.28	18.23	45.47	43.55	10.98	13.97	27.79	58.24
RHCNP	< 10	56.82	24.86	18.33	48.07	46.32	5.60	22.82	37.69	39.49
RHC@FU	< 10	61.01	24.36	14.64	45.70	43.97	10.33	17.45	44.93	37.62
RHC@AFU	< 10	45.89	30.93	23.18	33.62	56.93	9.45	28.97	50.91	20.12

Table S2. Distribution coefficient (K_d) and selectivity coefficient (α) under competitive adsorption conditions.

Adsorbent	K_d(Cd)	K_d(Zn)	α(Cd/Zn)	α(Zn/Cd)
RHC	1.16	0.86	1.34	0.74
RHCNP	1.54	1.23	1.25	0.80
RHC@FU	1.30	1.07	1.22	0.82
RHC@AFU	1.24	1.00	1.25	0.80

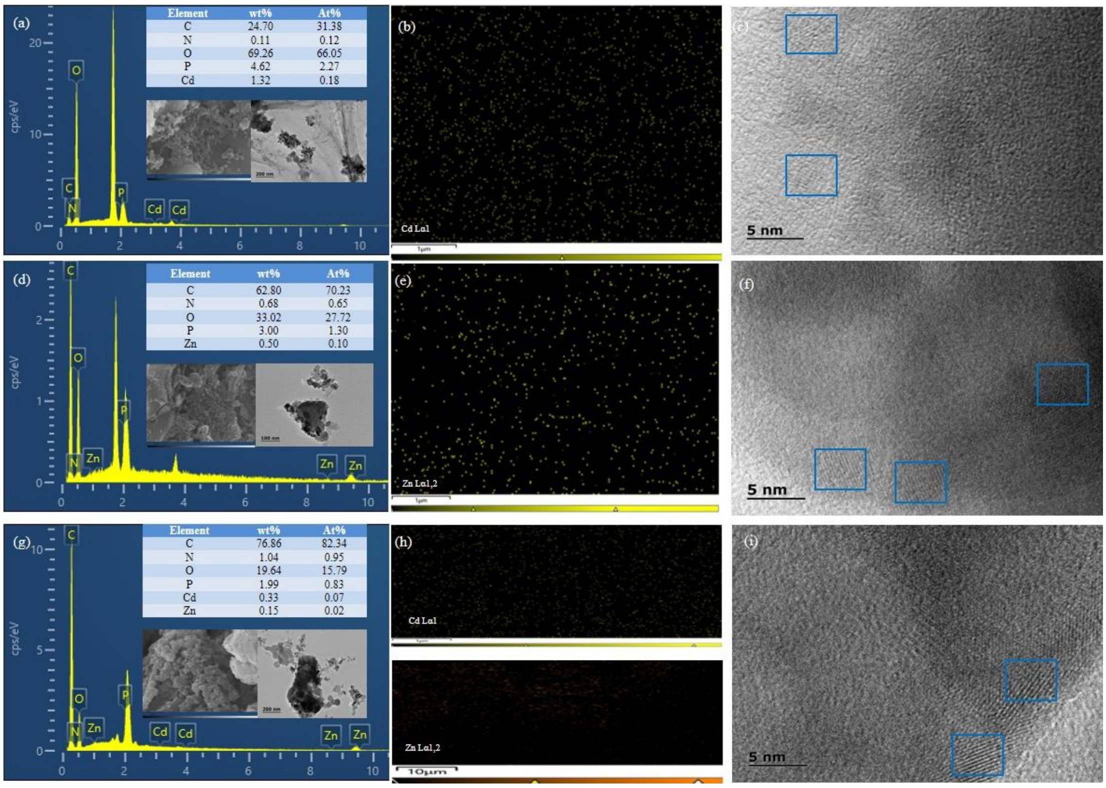

Figure S2. (a, b) EDS spectra of RHCNP after adsorption with Cd^{2+}; (d, e) RHCNP after adsorption with Zn^{2+}; (g, h) RHCNP after adsorption with Cd^{2+} and Zn^{2+}; TEM images of (c) RHCNP after adsorption with Cd^{2+}; (f) RHCNP after adsorption with Zn^{2+}; (i) RHCNP after adsorption with Cd^{2+} and Zn^{2+}.

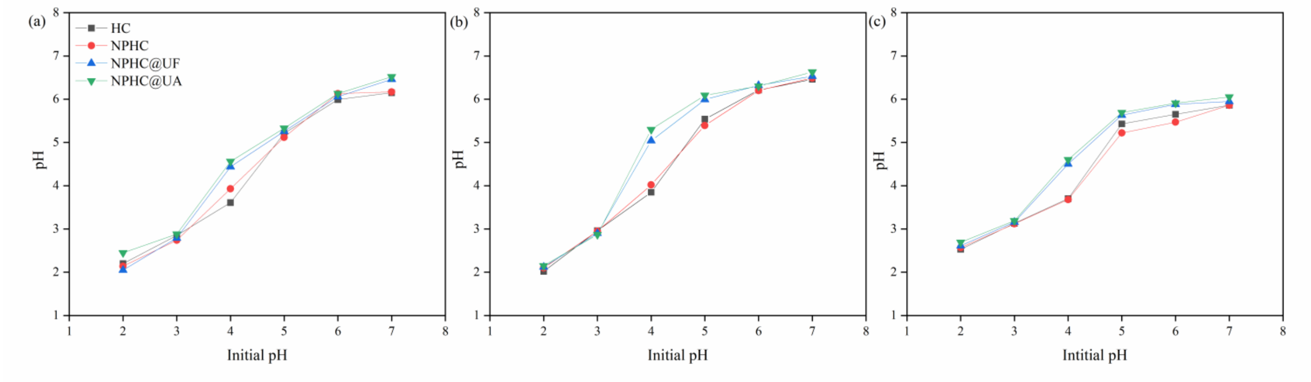

Figure S3. The pH of the solution before and after adsorption ((a) single Cd^{2+} system, (b) single Zn^{2+} system, and (c) binary metal system).

Table S3. Comparison of comprehensive Cd^{2+} and Zn^{2+} adsorption performance by different adsorbents.

Adsorbent	Adsorption capacity	Stability Capacity	Preparation procedure	Environmentally friendly properties	Recyclability potential	Material affordability	Ref.
1. Palygorskite	★★	★★★★	★★★★	★★★	★	★	[4, 5]
2. Zeolite	★	★★★★	★★	★★★	★★	★★★	[6]
3. Sludge	★★	★★★★	★★★	★★	★	★★★★	[7, 8]
4. Magnetic Graphene	★★★★	★★	★★	★★	★★	★	[9, 10]
5. PVA Hydrogel Beads	★	★★	★★★	★★	★★★	★★	[11]
6. Biochar	★★★	★★★	★★	★★★★	★★	★★★★	[12]
7. RHCNP	★★★	★★★	★★★	★★★★	★★	★★★★	This work

More ★ represent a better performance of this index. The number of ★ under each index is based on the following text survey comparison.

Reference

1. Boparai, H.K.; Joseph, M.; O'Carroll, D.M. Kinetics and thermodynamics of cadmium ion removal by adsorption onto nano zerovalent iron particles. J. Hazard. Mater. **2011**, 186, 458–465.
2. Li, K.; Wang, Y.; Huang, M.; Yan, H.; Yang, H.; Xiao, S.; Li, A. Preparation of chitosan- graft -polyacrylamide magnetic composite microspheres for enhanced selective removal of mercury ions from water. *J. Colloid Interface Sci.* **2015**, *455*, 261–270, https://doi.org/10.1016/j.jcis.2015.05.043.
3. Deng, J.; Liu, Y.; Liu, S.; Zeng, G.; Tan, X.; Huang, B.; Tang, X.; Wang, S.; Hua, Q.; Yan, Z. Competitive adsorption of Pb(II), Cd(II) and Cu(II) onto chitosan-pyromellitic dianhydride modified biochar. *J. Colloid Interface Sci.* **2017**, *506*, 355–364, https://doi.org/10.1016/j.jcis.2017.07.069.
4. Wang, H.; Wang, X.; Ma, J.; Xia, P.; Zhao, J. Removal of cadmium (II) from aqueous solution: A comparative study of raw attapulgite clay and a reusable waste–struvite/attapulgite obtained from nutrient-rich wastewater. *J. Hazard. Mater.* **2017**, *329*, 66–76.
5. Jiang, Y.; Sun, Y.; Pan, J.; Zhang, Y. Use of dewatered sludge as microbial inoculum of a subsurface wastewater infiltration system: effect on start-up and pollutant removal. *Water SA* **2017**, *43*, 595, https://doi.org/10.4314/wsa.v43i4.07.
6. Li, X.; Shi, J. Simultaneous adsorption of tetracycline, ammonium and phosphate from wastewater by iron and nitrogen modified biochar: Kinetics, isotherm, thermodynamic and mechanism. *Chemosphere* **2022**, *293*, 133574.
7. Siswoyo, E.; Mihara, Y.; Tanaka, S. Determination of key components and adsorption capacity of a low cost adsorbent based on sludge of drinking water treatment plant to adsorb cadmium ion in water. *Appl. Clay Sci.* **2014**, *97-98*, 146–152, https://doi.org/10.1016/j.clay.2014.05.024.
8. Du, X.; Cui, S.; Fang, X.; Wang, Q.; Liu, G. Adsorption of Cd(II), Cu(II), and Zn(II) by granules prepared using sludge from a drinking water purification plant. *J. Environ. Chem. Eng.* **2020**, *8*, 104530, https://doi.org/10.1016/j.jece.2020.104530.
9. Ain, Q.-U.; Farooq, M.U.; Jalees, M.I. Application of magnetic Graphene oxide for water purification: Heavy metals removal and disinfection. *J. Water Process. Eng.* **2020**, *33*, 101044.

10. Bao, S.; Yang, W.; Wang, Y.; Yu, Y.; Sun, Y. One-pot synthesis of magnetic graphene oxide composites as an efficient and recoverable adsorbent for Cd(II) and Pb(II) removal from aqueous solution. *J. Hazard. Mater.* **2020**, *381*, 120914, https://doi.org/10.1016/j.jhazmat.2019.120914.
11. Li, T.; Liu, X.; Li, L.; Wang, Y.; Ma, P.; Chen, M.; Dong, W. Polydopamine-functionalized graphene oxide compounded with polyvinyl alcohol/chitosan hydrogels on the recyclable adsorption of cu(II), Pb(II) and cd(II) from aqueous solution, *J. Polym. Res.* **2019**, *26* 281.
12. Cairns, S.; Chaudhuri, S.; Sigmund, G.; Robertson, I.; Hawkins, N.; Dunlop, T.; Hofmann, T. Wood ash amended biochar for the removal of lead, copper, zinc and cadmium from aqueous solution. *Environ. Technol. Innov.* **2021**, *24*, 101961, https://doi.org/10.1016/j.eti.2021.101961.
